# Edinburgh Royal Infirmary

**Published:** 1829-10-01

**Authors:** 


### EDINBURGH ROYAL INFIRMARY.

### I. A CASE OF EXTRAORDINARY HYPERTROPHY OF THE HEART.

Jane M'Lean, æt. 20, admitted 12th May, 1829, under Dr. Duncan.

*Symptoms on Admission.* Has a frequent and severe cough, attended with a profuse watery expectoration, tinged with blood. The respiration becomes hurried and distressing, even to a sense of suffocation, when she inclines to the horizontal posture. The action of the heart is heard over a larger space of the surface of the chest than natural, and the pulsations are stronger than natural. Palpitations are excited by trifling causes, and are generally followed by a fit of coughing. Pulse 100, moderately full, rather resisting, and occasionally intermitting. Has severe head-ache, which is aggravated very much by coughing; the face is very pale; has general œdema; the bowels are open; the urine is scanty.

*History of preceding Symptoms, &c.* About two years ago had fever; after which, palpitations arose, and which palpitations have since re-appeared on any exertion. About two months ago the cough and difficulty of breathing commenced—since then, have continued to increase in severity. The œdema and blood in sputa were only noticed one month ago. She was in the infirmary last Winter, complaining of head-ache and palpitations, and was dismissed somewhat relieved.

*Symptoms and Treatment after Admission.* ℞. Antim. tartarizati, gr. ij. Aquæ font. ℥viij. Tinct. opii, ʒjss. Syrupi, ℥ss. M. capiat ℥ss. tertia quaque horâ; habeat haustum anodynum horâ somni.

13th. Had a bad night; vomited; symptoms continue. Sumat pulv. digitalis, gr. j. omni horâ ad sextam vicem nisi supervenirit vomitus. ℞. Spt. ætheris nitrosi, ℥ss. Syrupi, ℥j. Aquæ font. ℥vj. M. capiat ℥ij. ter in die.

14th. Sickness was produced by three powders; cough less troublesome; the urine scanty; pulse 100, full and regular. Sumat tinct. scillæ, guttas xij. ter dedie ex aqua font; habeat pil. hydrargyri j. omni mane, et intermittantur medicamenta heri prescripta. It is unnecessary to pursue the diurnal details. She lingered till the sixth June, when she expired.

*Post-Mortem Examination.* There is a general œdema, but not to any great degree. The lower part of the right side of the chest sounds duller than natural on percussion. On elevating the sternum and cartilages of the ribs, the heart is seen to occupy a much larger space than usual, reaching from about the second rib to between the seventh and eighth. In each side of the chest there is about ℔jss. of serum, and in the abdomen about ℔s iv. The diaphragm is thrust more into the abdomen than natural, and, consequently, so is the liver. The lower parts of the right lung are much diseased; being of a dusky red or brown colour, and perfectly hepatized: the other parts are not so much altered; for though they are firmer than natural, the areolar structure can still be recognized, and they crepitate on pressure. The left lung is in no particular so changed as the right lung; but is of a greyish white colour, approaching, according to Dr. Duncan, to the character of the grey hepatization; it is likewise more filled with serum than natural. The heart weighed 32 ozs. that is, 22 ozs. more than the ordinary weight; and it was seven inches in length and about five inches in breadth. The fibrous and loose portion of the bag of the pericardium is, in this case, in all places adherent to that thinner portion which closely invests the heart itself. This adhesion between the two layers of the pericardium is by means of a loose cellular tissue, which is infiltrated with serum. The auriculo-ventricular valves are diseased; more especially the mitral valve, which is thickened, contracted, and has some calcareous deposition on it. The pulmonary veins are exceedingly small. The ascending, the transverse arch of, and the descending, aorta are smaller in caliber, and have thinner coats than natural.

### II. LARYNGITIS—TRACHEOTOMY.

*Case* 2. Mary Taylor, æt. 28, admitted May 23d, at 2, p.m. under Dr. Duncan.

*Symptoms on Admission.* Complains of pain in the larynx and trachea, which is increased by external pressure. Inspiration is extremely difficult, causing pain in the larynx, and producing a loud croupy sound. Expiration is free; expectoration difficult; sputa scanty and viscid. She has a harsh and painful cough; and very great anxiety expressed in the countenance. Pulse 100, and smaller than natural. Skin is moist; bowels are open. Has a deep ulcer on the uvula, and a superficial one on the fauces.

*History of preceding Symptoms, &c.* The ulcers on the uvula and fauces have existed for seven months. At intervals since their appearance she has been seized with difficult deglutition, and inspiration likewise has at times been difficult in some degree, but never before the present attack has it been distressing. For the last fortnight she has had a sense of soreness about the fauces, and deglutition and inspiration, during the same period, have been painful in some degree; but the extreme difficulty of breathing under which she now labours, appeared only the day before her admission to the infirmary. It came on suddenly, and quickly became aggravated to its present intensity.

*Symptoms and Treatment after Admission.* In the evening the symptoms became more aggravated. Applicentur hirudines, xij. faucibus externis et postea cataplasmta, et ℞. Calomælanos, gr. iv. Pulv. antimonialis, gr. vi. M. statim. sumendus.

24th. Inspiration is much more difficult and painful; and, during which, the soft parts above the clavicles are sucked in. She has slept none; headache is very distressing; expectoration difficult; pulse 100, rather small; tongue moist; bowels open; countenance anxious; face flushed; voice nearly lost. ℞. Sulph. cupri, gr. v. Aq. font, ℥ij. solve et sumat statim; post emeticum bibat aquæ tepidæ, ℔j.; emetio finito admoveatur emplast. epispasticum collo; et ℞. Hydr. submur. gr. x. Opii, gr. ij. M. fiant pilulæ vj. sumat duas omni bihorio.

25th. The dyspnœa was relieved for some time by the emetic, but has since returned. She sits almost upright in the bed; and inspiration is much more difficult, incomplete, and painful, than it was yesterday. The obstruction is referred by the patient distinctly to the glottis. The face is flushed; the eyeballs project; and there is almost perfect aphonia. The pulse intermitting. As it was evident that tracheotomy would give the only remaining chance of recovery to the patient, its performance was decided on, and immediately executed. *The operation.* Mr. Liston commenced by making a perpendicular cut of about an inch and a half in length through the integuments over the trachea, commencing below the cricoid cartilage; he then slit up two or three rings of the trachea, and introduced a curved tracheal tube of rather large caliber, which was fixed in its situation by means of tape passed through the rings at the external end of the instrument, and tied round the patient's neck. Instantaneous relief was obtained by the operation; for, after two or three convulsive expirations, (caused by some drops of blood entering the trachea from the wound,) she breathed with perfect ease. She got an anodyne at bed-hour, which was repeated for three successive nights. A very large quantity of mucus was expectorated for the first three days after the operation. The ulcers of the uvula and fauces have healed by the application of nitrate of silver; but with an almost complete destruction of the former appendage. During the process of cure a thick piece of bone came away, which some of the surgeons think to be an ossified portion of the thyroid cartilage. A smaller tube has lately been substituted for the one first introduced ; the voice is much restored, and it is hoped that the artificial opening may soon be dispensed with, and the breathing established through the natural passages.

*Remarks*. This case well exemplifies the utility of tracheotomy, when not long deferred, in cases of threatened suffocation from inflammation of the larynx. No irritation, no cough, no sense of choaking, was, in this case, produced by the canula.

### XXXIX.

### EDINBURGH ROYAL INFIRMARY.*

### I. TEMPORAL ANEURISM FROM ARTERIOTOMY.

Small aneurismal tumours of the temporal artery are by no means unfrequent after arteriotomy, but if treated in an early stage they are almost always curable by pressure. It would seem that the divisions of the temporal, and indeed of the other arteries of the scalp, are peculiarly liable to dilatation, whereas, in the extremities, especially the lower, the veins are most commonly affected. These two sets of vessels are placed under somewhat similar circumstances with regard to the circulation, which is, cæteris paribus, more feeble in both, than in arteries or veins elsewhere. Putting, however, hypothesis aside, we shall notice a case of aneurism of the temporal artery, where compression failed, and excision of the tumour was performed.

The patient was a young man, 28 years of age, admitted on the 8th of December, with aneurism of the anterior branch of the temporal artery, the size of a nut, and of livid colour. It had followed arteriotomy practised about three weeks previously. Compression, by means of a hard compress, being tried without success, the tumour, with a small portion of the surrounding integuments, was removed by an elliptical incision, the inferior extremity of the artery tied, and the wound dressed in with lint. Healthy granulations followed, and on the 2d of January the patient was dismissed cured.

Dr. Ballingall believes that, in a case like this, division of the artery above and below the tumour, with subsequent compression, would be equally efficient with excision, and speedier in its results. We may mention in this place the particulars of a case of varicose veins of the right leg, successfully treated by caustic in the manner recommended by Mr. Mayo. The affection was of six months' standing, the veins below the knee much swollen, and attended with a good deal of œdema. Repose and laxatives having been premised, Dr. Ballingall applied the caustic paste over the vein above the knee, without any very sanguine hopes of success. The paste was allowed to remain for seven hours, then removed, and an emollient poultice applied. In "ten *or* fourteen" days the eschar separated, and the trunk of the vein lay exposed to the extent of upwards of an inch in the bottom of the wound; it could also be felt hard and swollen for an inch below, and two or three above the site of the eschar. The denuded portion, although black, did not slough, healthy granulations gradually sprang up and covered it over, and in something less than a month from his admission the patient left the house. The sore at this time was cicatrizing rapidly; "and, what was remarkable, although the principal dilatation of the vein immediately below the knee did not appear to be much diminished while the limb was at rest, yet no sooner was it put in motion than the blood began to circulate freely through the collateral veins, and the swelling to disappear."

We fear that the prospect of permanent relief, for *cure* there was none, is not very brilliant in the present case. Our opinions on the caustic treatment of varix have already been expressed, and having hitherto seen no reason for a change, we need not repeat them again. By the bye, Mr. Mayo is not fairly the inventor of the method, such as it is, for we learn from an eminent surgeon of this town, that he has seen the caustic potass employed, and made use of it himself, many years ago. In his experience it was safe, but on the whole *not* successful.

### II. DISEASE AND REMOVAL OF THE BONES OF THE CRANIUM.

Every surgeon must have witnessed the comparative impunity with which large portions of the cranial bones may be removed in cases of chronic disease. We have seen nearly the whole of one parietal bone taken away by the trephine and lever in a case of scrofulous caries, and that with scarcely an untoward symptom. Dr. Ballingall refers his readers on this point to the remark-

* Dr. Ballingall's Clinical Lecture.

able case detailed by Saviard, of a woman in the Hôtel Dieu, "who underwent successive exfoliations of the cranial bones to such an extent that the pieces, when put together, resembled the skull-cap as it is sawn off in dissections." Nature seems to make up her mind to suffer an incredible amount of injury provided it be gradually inflicted. The following are interesting cases, because they are of frequent occurrence in practice.

*Case* 1. James Thorn, ætatis 13, admitted on the 10th of December under the care of Mr. Liston, with an ulcerated opening in the scalp immediately over the junction of the parietal bones towards their back part. Across this opening there passed a small bridge of integument, so as to divide it into two, and at the bottom of the sore the matter was distinctly seen rising and falling in synchrony with the pulsation of the brain. On introducing a probe, the bone was felt bare for a considerable distance round the wound on all sides; the health had not suffered; there was no pain of head; pupils quite sensible, but rather dilated; tongue white; appetite good; pulse 74, and somewhat irregular. He stated that two years and a half ago he received a blow on the head from a stone—that a swelling formed which opened in three days after the accident, and gave issue to a small quantity of blood—and that, since that time, several portions of bone had come away.

On the 14th, an incision was made in the scalp, and the bone discovered extensively bare, but nothing further was done until the 26th, when a circular portion of bone was removed, in order to give a free exit to the matter. No bad symptoms followed, the wound assumed a healthy aspect, and about the middle of January the patient was dismissed, with instructions to return and show himself occasionally. Upon the last examination the wound was cicatrizing rapidly, and the boy's general health was good.

The next is a more severe example of these affections, but it offers equally, if not more decisive proof of the powers of art in arresting their progress.

*Case* 2. Henry Lee, æt. 13, was admitted on the 23d of December, with an opening the size of a shilling in the scalp, situated over the junction of the occipital with the parietal bones, and the sagittal suture. The bone was black and bare for a considerable distance round; the discharge was thin and offensive; no pain in the head; pupils dilated, but perfectly sensible; pulse 104; tongue moist; health and appetite good. Over the sternum was a fistulous opening also leading down to bare bone. Twelve months previously he had received a blow upon the head, and an abscess ensued which was opened a few days after the accident. Pain in the head succeeded this, and in about a week he had a fit, and this was followed by a discharge of matter, which in its turn relieved the pain. After this he had a great number of fits, all of which were followed by a discharge of pus. Since the 17th of September he had had no fit. The abscess in the sternum had formed and was opened much about the same time as that on the head.

On the 23d, a crucial incision was made, and a piece of bone, the whole thickness of the cranium, found loose and removed. No bad symptom whatever succeeded, and on the 27th, he was ordered animal food, and a few days afterwards four ounces of wine daily. From this time his cure went on progressively, and, on the 10th of January, he was made an out-patient. The fistulous opening over the sternum was touched with the actual cautery, and ultimately healed.

"Both these cases afforded examples of the beneficial interference of art in removing large portions of the cranial bones when in a state of disease. The contrast between such cases and those of recent injury is very remarkable, for, while in the latter every experienced surgeon dreads the extensive exposure of the dura mater, yet he knows that in cases like those I have just detailed, where the dura mater has been for some time detached from the interior of the skull, and has become covered with granulations, the carious or necrosed bone may be removed with freedom, and often with advantage."

